# Tolerability of facial electrostimulation in healthy adults and patients with facial synkinesis

**DOI:** 10.1007/s00405-020-05818-x

**Published:** 2020-01-24

**Authors:** Gerd Fabian Volk, Jovanna Thielker, Martin Christian Möller, Daniela Majcher, Valeria Mastryukova, Carolin Susann Altmann, Christian Dobel, Orlando Guntinas-Lichius

**Affiliations:** 1 Department of Otorhinolaryngology, Jena University Hospital, Am Klinikum 1, 07747 Jena, Germany; 2 Facial Nerve Center Jena, Jena University Hospital, Jena, Germany; 3 Present Address: Institute of Anatomy I, Jena University Hospital, Jena, Germany

**Keywords:** Electrostimulation, Mimetic muscles, Facial movements, Facial palsy, facial paralysis

## Abstract

**Purpose:**

To evaluate optimal stimulation parameters with regard to discomfort and tolerability for transcutaneous electrostimulation of facial muscles in healthy participants and patients with postparetic facial synkinesis.

**Methods:**

Two prospective studies were performed. First, single pulse monophasic stimulation with rectangular pulses was compared to triangular pulses in 48 healthy controls. Second, 30 healthy controls were compared to 30 patients with postparetic facial synkinesis with rectangular pulse form. Motor twitch threshold, tolerability threshold, and discomfort were assessed using a numeric rating scale at both thresholds.

**Results:**

Discomfort at motor threshold was significantly lower for rectangular than for triangular pulses. Average motor and tolerability thresholds were higher for patients than for healthy participants. Discomfort at motor threshold was significantly lower for healthy controls compared to patients. Major side effects were not seen.

**Conclusions:**

Surface electrostimulation for selective functional and tolerable facial muscle contractions in patients with postparetic facial synkinesis is feasible.

**Electronic supplementary material:**

The online version of this article (10.1007/s00405-020-05818-x) contains supplementary material, which is available to authorized users.

## Introduction

In severe facial nerve paralysis with degeneration of nerve fibers (axonotmesis or neurotmesis), spontaneous regeneration or regeneration after nerve reconstruction typically is disordered and leads in the chronic phase to altered patterns of muscle contraction and postparetic synkinesis. This situation has to be distinguished from the much rarer situation of a patient without any regeneration and chronic muscle denervation. As in the acute phase of facial paralysis, the application of facial electrostimulation in patients with postparetic synkinesis is controversial [1]. The aim of electrostimulation in the postparetic chronic phase is to selectively induce and improve functionally relevant movements like eye closure or smiling. However, there is a lack of studies systematically analyzing optimal stimulation parameters for facial surface electrostimulation [2]. Related to the question of optimal stimulation from a functional point of view, is the sometimes asserted that electrostimulation in the face is limited by its low tolerance to electricity, especially in patients with facial palsy. From surface facial nerve mapping to detecting the course of peripheral facial nerve branches, we only know that the stimulation thresholds are higher in patients with postparetic facial synkinesis than in healthy persons [3]. Therefore, it is necessary to combine investigations on the efficacy of facial electrostimulation with pain or comfort ratings of the patients [4].

Highly topical, optimal and tolerable electrostimulation is immanent to future bionic applications for facial reanimation by an active implantable device. A bionic facial device will only be approved by authorities and accepted by patients if the electrostimulation can be applied without any discomfort or painful sensation for the patients.

The present prospective clinical study was performed to analyze systematically the optimal parameters for surface electrostimulation of the face with innervated facial musculature. Denervated musculature was not addressed. The aim was to define stimulation parameters allowing selective facial muscle contractions without discomfort. The study was conducted in two parts. In the first study, two different pulse waveforms were analyzed in healthy participants to define the design of the subsequent second study. Here, a defined set of pulse durations and positions in the face were analyzed in healthy participants in comparison to patients with postparetic facial synkinesis.

## Materials and methods

### Study design and setting

This prospective interventional study was performed in 2017 at the Department of Otorhinolaryngology, authors blinded. Approval for the study was obtained through the local institutional review board and informed consent was obtained from all study participants. The investigation had two parts: (1) In the first study, two different waveforms of the pulses were compared only in healthy controls. The effects of surface facial electrostimulation using pulses with rectangular waveform were compared to pulses with triangular waveform to decide if a rectangular or triangular waveform should be used for the second study. (2) Based on the results, only pulses with rectangular waveform were used for the subsequent second study. Here, the effects of electrostimulation in healthy participants were compared to patients with aberrant reinnervation after facial palsy and subsequent postparectic facial synkinesis.

The first study investigated 48 healthy controls (20 females, median age: 45 years). Inclusion criteria were: no history of facial palsy or any other neurological disease, no other chronic disease. The stimulation was randomly performed on the left side or the right side in half of the controls, respectively. In the second study, another group of 30 healthy controls (8 females, median age: 49 years) with the same inclusion criteria were recruited. The additional group of patients consisted of 30 participants (22 females, median age: 51 years) with postparetic facial synkinesis as the result of unilateral peripheral facial palsy of different origin (idiopathic, herpes zoster, posttraumatic, postsurgical). The time interval between onset of the palsy and inclusion into the study was at least 12 months. The patients had postparetic synkinesis for at least 4 months. Postparetic synkinesis was confirmed by clinical and electromyographical examination. The patients were stimulated on the affected side.

### Transcutaneous facial electrostimulation

Electrostimulation was performed using a standard electrostimulation device (Galva 4a, Zimmer Medizin Systeme GmbH, Neu-Ulm, Germany). For stimulation, round stimulation electrodes (diameter: 32 mm; Krauth + Timmermann GmbH, Hamburg, Germany) were used. A ground electrode (56 × 56 mm, Zimmer Medizin Systeme GmbH, Neu-Ulm, Germany) was fixed on the skin in the neck. All participants were examined in a sitting position. The stimulation took place at three defined positions (Supplemental Fig. 1): (P1) Midline between lateral corner of the eye and base of the helix for contraction of the orbicularis oculi muscle; (P2) below the zygomatic arch for zygomatic muscle contraction; and (P3) on the level of the angle of the mouth anterior to the masseter muscle for orbicularis oris muscle contraction. The sequence of the positions for electrostimulation was counterbalanced across participants. Single monophasic pulses were used. A sequence of fixed pulsation durations (0.1, 0.5, 1, 10, 50, 100, 500 ms) was tested. As a result of the first study, we refined the gradation of the pulsation durations (0.1, 0.5, 1, 2, 5, 10, 50, 100, 500, 1000 ms) for the second study. The stimulation at each stimulation point started with 0.1 mA and was stepwise increased in 0.1 mA steps. The following parameters were evaluated: (1) motor twitch threshold: the point at which a facial muscle contraction was observed, (2) tolerability threshold: the point at which the participant wanted to stop the stimulation because of discomfort or when the maximal amplitude (40 mA) was reached. The tolerability threshold was reached in some cases instantly at the first stimulation at the lowest threshold, i.e. the tolerability threshold was lower than the motor threshold. Additionally, the participant rated the felt discomfort after electrostimulation on a numeric rating scale (NRS) from 0 (no discomfort) to 10 (maximal discomfort). This was used to record (3) discomfort at motor threshold, and (4) discomfort at tolerability threshold. Finally, all side effects were noted. The participants were actively asked for the following side effects after each electrostimulation: light flashes, toothaches, metallic taste. If occurred, the participants were asked to qualify the side effect as mild, tolerable, intolerable. Furthermore, the participants were observed for unintended masseter muscle or shoulder muscle contractions. If occurred, the participants were also asked to qualify the side effect as mild, tolerable, intolerable.

**Fig. 1:**
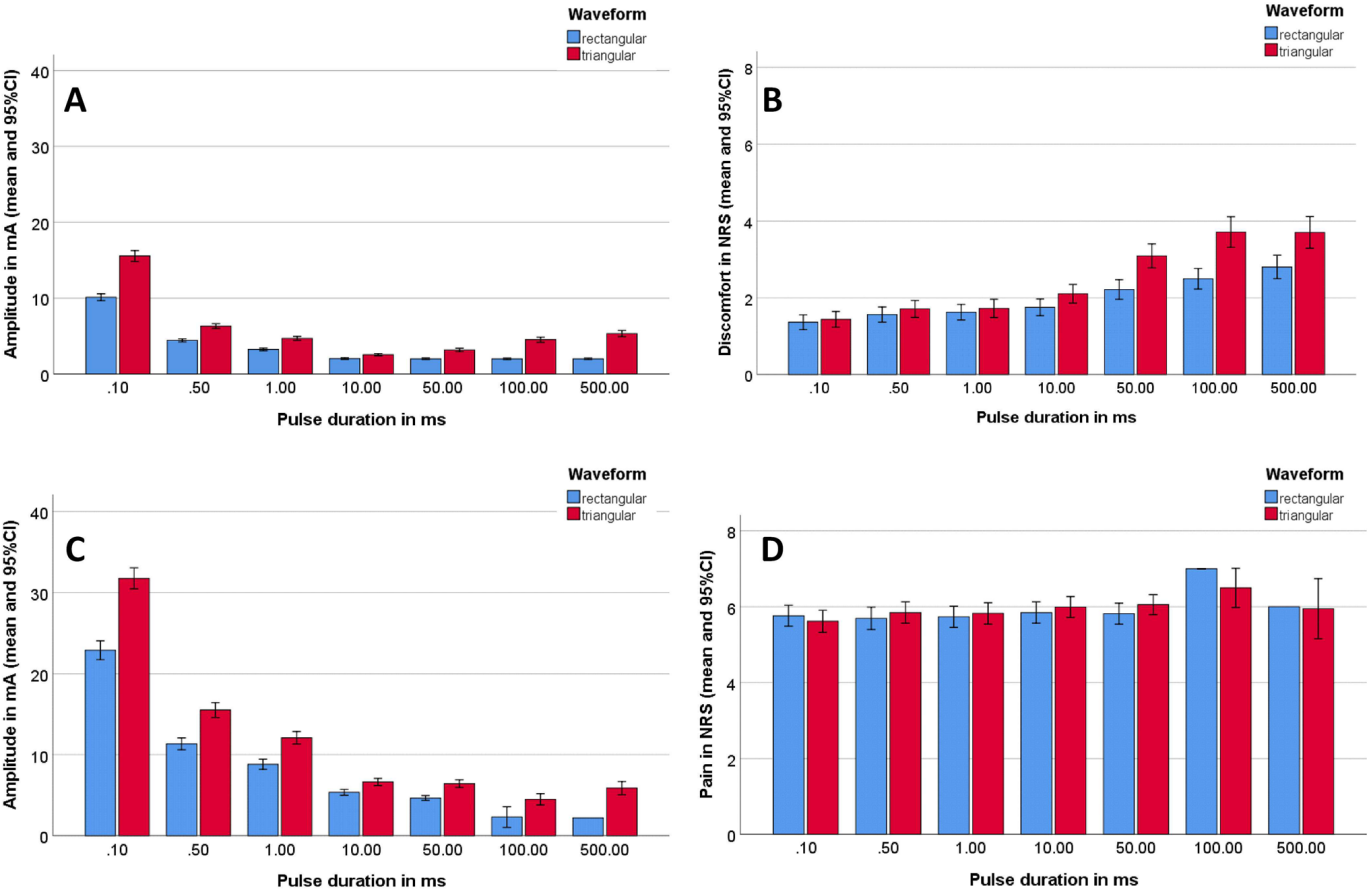
First study: comparison of rectangular (blue) and triangular pulses (red) in healthy adults (*N* = 48) at the different pulse durations (*x*-axis) averaged for all three stimulation sites. **a** Motor threshold; **b** discomfort at motor threshold; **c** tolerability threshold; **d** discomfort at tolerability threshold. *NRS* numeric rating scale

### Statistics

All outcome variables were analyzed with IBM SPSS statistics software (Version 25; IBM, New York) for medical statistics. Data are presented as frequencies or mean ± standard deviation (SD) if not otherwise indicated. Amplitude and discomfort threshold as primary outcome parameters were analyzed using repeated measures analysis of variance (rmANOVA). Bonferroni corrected pairwise *t*-tests were used for post hoc comparisons. Gender and side (stimulation left or right side) were between-subject factors. Pulse duration up to 50 ms (not 100 ms and 500 ms, because the tolerability threshold was not reached at these pulse durations), stimulation site, and waveform of the stimulus were within-subject factors in each rmANOVA in the first study. In the second study, gender and health status (healthy controls, patients) were between-subject factors. Pulse duration up to 50 ms (not 100 ms, 500 ms and 1000 ms, because the tolerability threshold was not reached at these pulse durations), and *stimulationsite* were within-subject factors in each rmANOVA. The significance level was set at *p* < 0.05.

## Results

### First study: comparison of rectangular and triangular pulses in healthy controls

Average amplitude levels at motor threshold and discomfort at motor threshold, as well as discomfort at tolerability threshold and discomfort tolerability threshold averaged over the three stimulus positions P1, P2 and P3 are shown in Fig. 1. The complete dataset is summarized in Supplemental Table 1. Up to a pulse duration of 10 ms, the tolerability threshold was always higher than the motor threshold for rectangular and also for triangular pulses. At 50 ms, non-tolerable discomfort sensation was reported by one subject before the motor threshold was reached. At 100 ms and 500 ms, this was the case for 73–98% and 22–98% of the participants.

The visual impression of the results was confirmed statistically: a rmANOVA was performed for each of the four primary outcome factors and revealed the following: The amplitude at motor threshold was significantly lower for P1 than for P2 and P3 (main effect stimulation site *F*[2, 88] = 27.149, *p* < 0.001, *η*^2^ = 0.382), decreased with longer pulse duration (main effect pulse duration *F*[4, 176] = 1054.567, *p* < 0.001, *η*^2^ = 0.960), and was higher for triangular than for rectangular pulses (main effect waveform *F*[1, 44] = 434.00, *p* < 0.001, *η*^2^ = 0.908). Gender and side had no influence on the motor threshold. The average threshold for rectangular pulses was 4.4 mA (95% confidence interval [CI] = 4.1–4.6) and for triangular pulses 6.4 mA (95% CI 6.0–6.9; *p* < 0.0001). The same effects were seen in the ANOVA for the amplitude at tolerability threshold. The motor threshold was significantly lower for P1 than for P2 and P3 (main effect stimulation site *F*[2, 88] = 12.696, *p* < 0.001, *η*^2^ = 0.224), decreased with longer pulse duration (main effect pulse duration *F*[4, 176] = 832.581, *p* < 0.001, *η*^2^ = 0.950), and was higher for triangular than for rectangular pulses (main effect waveform *F*[1, 44] = 357.578, *p* < 0.001, *η*^2^ = 0.890). Gender, and side had no influence on threshold (all *p* > 0.05). The average threshold for maximal tolerance for rectangular pulses was 10.7 mA (95% CI 9.6–11.7) and for triangular pulses 14.7 mA (95% CI 13.5–15.9; *p* < 0.0001).

The rmANOVA for the discomfort at motor threshold revealed that discomfort perception increased with longer pulse duration (main effect pulse duration *F*[4, 176] = 109.074, *p* < 0.001, η^2^ = 0.586), and discomfort was higher for triangular than for rectangular pulses (main effect waveform *F*[1, 44] = 17.650, *p* < 0.001, η^2^ = 0.286). Average discomfort at motor threshold (NRS) for rectangular pulses was 1.6 (95% CI 1.3–1.9) and for triangular pulses 1.9 (95% CI 1.6–2.3; *p* < 0.0001). Stimulation site, gender, and side had no influence. The rmANOVA for discomfort at the tolerability threshold revealed that none of the factors or combinations of the factors influenced discomfort at the tolerability threshold (all *p* > 0.05). Longer pulse duration was associated to more discomfort per mA stimulation amplitude (Supplemental Fig. 2). The effect seemed to be more pronounced for rectangular pulses.

**Fig. 2:**
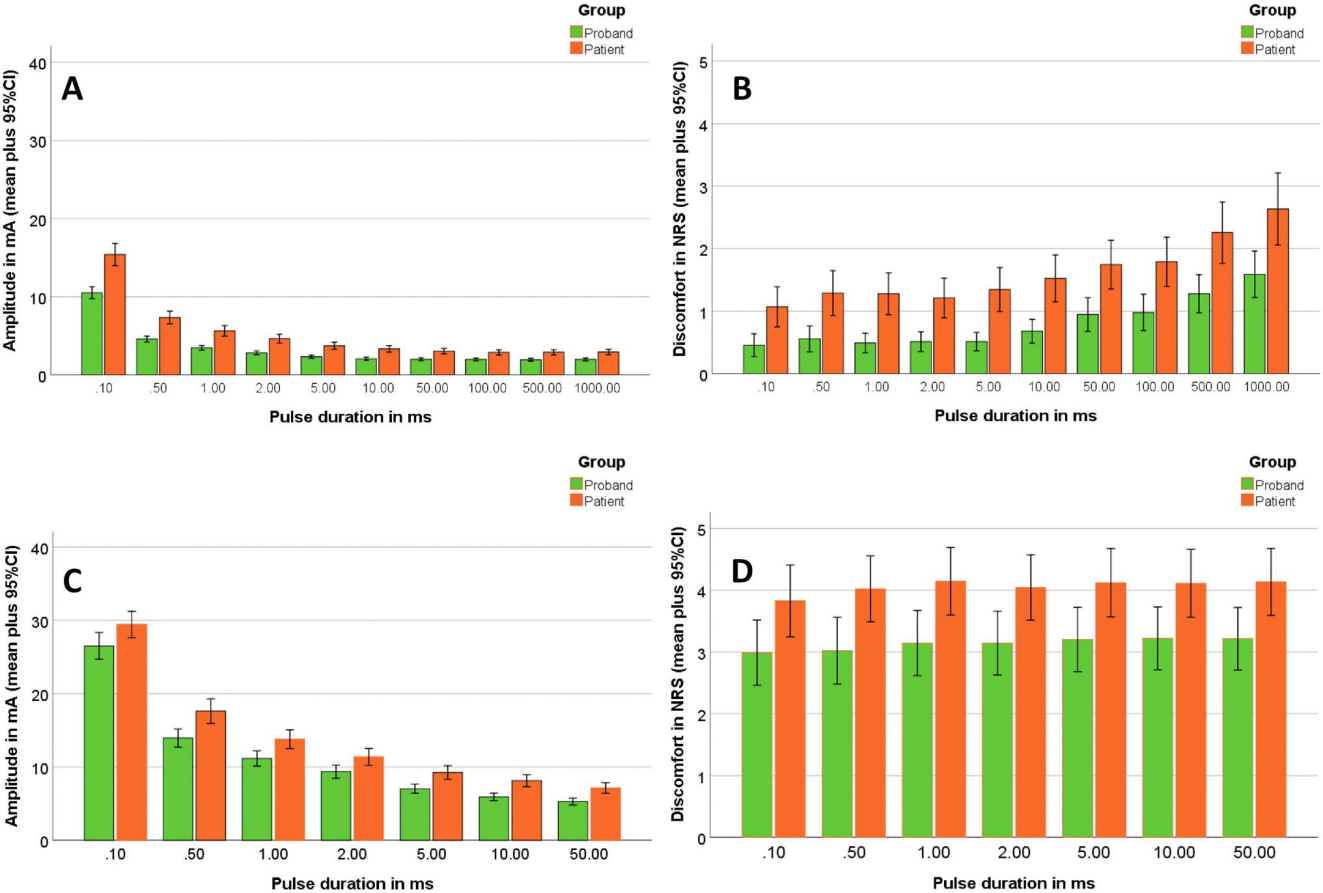
Second study: Comparison of healthy adults (*N* = 30; green) and patients with aberrant reinnervation after facial palsy (*N* = 30; orange) at the different pulse durations (*x*-axis) of rectangular pulses averaged for all three stimulation sites. At 100, 500 and 1000 ms, the tolerability threshold was directly reached at base values. Therefore, no values are shown for these three pulse durations. **a** Motor threshold; **b** discomfort at motor threshold; **c** tolerability threshold; **d** discomfort at tolerability threshold. *NRS* numeric rating scale

The side effects of electrostimulation are shown in Supplemental Table 2. Overall, side effects were more often seen for triangular than for rectangular pulses. The visual impression of light flashes during electrostimulation was by far the most frequent, but tolerable side effect. Tolerable weak light flashes were felt during 369 out of 1008 rectangular stimulations (36.6%) and during 393 of 1008 triangular stimulations (38.9%). Other relevant side effects in descending order of frequency were masseter muscle contraction, toothaches, a metallic taste, and shoulder muscle contraction. Light flashed occurred more often during electrostimulation at position P1 (eye region). Toothaches occurred most frequently at position P2 (cheek region). Masseter muscle contraction, shoulder muscle contraction, or metallic taste were seen most frequent at position P3 (oral region).

### Second study: comparison of electrostimulation in healthy controls to patients with facial palsy

Average amplitude levels at motor threshold and pain at motor threshold, as well as at tolerability threshold and discomfort tolerability threshold averaged over the three stimulus positions P1, P2 and P3 re shown in Fig. 2. The related data are summarized in Supplemental Table 3. Up to pulse duration of 50 ms, the tolerability threshold was always higher than the amplitude at motor threshold for healthy participants and patients. Beginning at 100 ms and at higher pulse duration, a non-tolerable discomfort perception directly occurred in 73–98% of the participants.

A rmANOVA was also performed for each of the four primary outcome factors in the second study. The motor threshold was significantly lower for P1 than for P2 and P3 (main *effectstimulationsiteF*[2, 102] = 4.785, *p* < 0.010, *η*^2^ = 0.086) and decreased with longer pulse duration (main effect pulse duration *F*[6, 306] = 231.815, *p* < 0.001, *η*^2^ = 0.820). Gender and side had no influence. There was a significant lower motor threshold for healthy controls than for patients. The average threshold for controls was 3.4 mA (95% CI 3.0–3.7) and for patients 5.2 mA (95% CI 4.3–6.0; *p* < 0.0001). The same effects were seen in the rmANOVA for the tolerability threshold. The motor threshold was significantly lower for P1 than for P2 and P3 (main effect stimulation site *F*[2, 96] = 4.330, *p* < 0.016, *η*^2^ = 0.083) and decreased with longer pulse duration (main effect pulse duration *F*[6, 288] = 296.281, *p* < 0.001, *η*^2^ = 0.861). Gender and side had no influence. There was also a significant lower motor threshold for healthy controls than for patients (main effect health status; *p* = 0.025). The average tolerability threshold for healthy controls was 11.3 mA (95% CI 9.9–12.8) and for patients 13.8 mA (95% CI 12.8–15.7; *p* = 0.044).

The rmANOVA for the discomfort at motor threshold in the second study revealed that discomfort perception increased with longer pulse duration (main effect pulse duration *F*[6, 306] = 4.569, *p* < 0.001, *η*^2^ = 0.082). Average discomfort at motor threshold (NRS) for healthy controls was 0.8 (95% CI 0.5–1.1) and for patients 1.6 (95% CI 1.0–2.2; *p* = 0.015). Stimulation site, gender, and *side* had no influence. The rmANOVA for discomfort at the tolerability threshold revealed that none of the factors or combinations of the factors had influence on discomfort at the tolerability threshold (all *p* > 0.05). Discomfort at average tolerability threshold (NRS) for healthy controls was 3.1 (95% CI 2.2–4.0) and for patients 4.1 (95% CI 3.1–5.0; *p* = 0.144), i.e. was not significantly different. As in the first study, longer pulse duration was associated to more discomfort per mA stimulation amplitude (Supplement Fig. 2). The effect was not different between healthy controls and patients, i.e. there was no interaction between the two factors.

The side effects of electrostimulation of the second study are shown in Supplemental Table 4. Again, tolerable light flashes were by far the most frequent side effect felt in 30% of the stimulation in controls as well as in patients. Overall, the frequency of felt side effects was not different for healthy controls and patients except for the sensation of metallic taste. Sensation of metallic taste was more frequently reported by the patients. Like in the first study, light flashes also occurred more often during electrostimulation at position P1 (eye region) in the second study. Toothaches occurred most frequently at position P2 (cheek region). Masseter muscle contraction and shoulder muscle contraction were seen most frequent at position P3 (oral region).

## Discussion

The presented analysis showed that surface electrostimulation of three functionally relevant facial regions is feasible (eye closure, smiling, and mouth closure) in awake healthy participants as well as in patients with postparetic facial palsy. Depending on the pulse duration, average stimulation intensities of about 7–9 mA (for very short pulses of 0.1 ms duration) to 1.7–3.0 mA (for pulses of 50 ms duration) were sufficient to elicit facial muscle contractions when using rectangular pulses which were better tolerated than triangular pulses. The range to the tolerability threshold was high: Depending on the pulse duration, average stimulation intensities of up to 24–30 mA (for very short pulses of 0.1 ms duration) to 5–7 mA (for pulses of 50 ms duration) were with tolerable discomfort. As it has already been shown for surface facial nerve mapping, the stimulation thresholds were higher in patients with postparetic facial synkinesis than in healthy persons [3]. This could be explained by a disturbed myelinization of the reinnervated axons causing a higher motor threshold. Nevertheless, this allows a wide range of electrostimulation to elicit a graduated facial function from mild to maximal muscle contractions.

Recently, Ilves et al. were able to produce an eyebrow raise, eye blink, smile, or lip pucker, respectively, with amplitudes of 2.2–3.6 mA for eye closure to about 5.1 mA for a functional smiling movement. In contrast, to the present study, they used biphasic rectangular pulses of 0.4 ms duration with a pulse repetition of 250 Hz for a duration of 80 ms [4]. This corresponds to the present subset of data for similar pulse duration of 0.5 ms, but with only one single monophasic rectangular pulse.

It is well known that facial discomfort—as in most other regions of the body—increases with the stimulus amplitude and becomes painful and intolerable with high amplitudes [4]. This was confirmed here. In addition, the present study showed that pulse duration had an important influence on this correlation. When using longer pulse duration, the increase of discomfort per increase of the stimulation amplitude was higher. Therefore, it might be reasonable for future studies and applications to work with rectangular pulses of less than 50 ms duration. We could not reveal significant differences of the tolerability between the different facial regions. Only the side effect of the perception of mild light flash sensation was seen more frequently with stimulation in the upper face. In a recent study, pain (as the most severe form of discomfort) occurred more at the mouth region and was less pleasant than in the forehead region [4]. A limitation of the present study was that only single pulses were used. It remains therefore unclear, if repeated pulses are more uncomfortable or even less uncomfortable due to a habituation effect. Furthermore, the patients had different causes of their facial palsy. We hypothesize (but cannot prove) that the underlying disease had no influence on the tolerability of facial electrostimulation.

Patients with postparetic facial synkinesis or weakened facial function after acute facial palsy are much more frequent than patients with chronic paralysis due to permanent denervation. Notably, patients with postparetic facial synkinesis have a reduced quality of life not different from patients with facial palsy with more motoric weakness [5, 6]. Recently, it has been shown that additional electrostimulation could produce effective eye blinks not only in completely paretic cases, but also in postparetic cases [2]. Using the parameters discussed, electrostimulation is tolerated well over longer time periods of 120 min at least for improved eye blink [7]. Therefore, we are convinced that it is worthwhile to develop electrostimulation concepts for patients with postparetic facial synkinesis to improve their quality of life.

Finally, functional electrical stimulation will be an immanent part of future implanted bionic devices to reanimate paretic facial muscles [8–10]. The stimulation protocol of the present study should become an optimal basis to be tested in the future diagnostic set-up for patients planned for facial muscle pacing and as stimulation parameters for the bionic device.

## Conclusions

This prospective cohort studies on single-pulse surface facial electrostimulation demonstrated that rectangular pulses were better tolerated than triangular pulses. Therefore, we recommend using the rectangular pulse form for further evaluation of optimal surface facial electrostimulation. Stimulation thresholds were higher in patients than in controls with healthy facial nerve and normal facial musculature. Nevertheless, surface facial electrostimulation was feasible and tolerable in a broad range of stimulation amplitudes in healthy participants and patients with postparetic synkinesis. These results may have implications on the planning of selective electrostimulation therapy or on the site of nerve stimulation of future bionic devices to restore facial nerve function.

## Electronic supplementary material

Below is the link to the electronic supplementary material.

Supplementary file1 (DOCX 678 kb)

Supplementary file2 (DOCX 238 kb)

Supplementary file3 (DOCX 71 kb)
