# Supplementary material for: Tolerability of facial electrostimulation in healthy adults and patients with facial synkinesis
Source: Eur Arch Otorhinolaryngol. 2020 Jan 24;277(4):1247–53. doi: 10.1007/s00405-020-05818-x (PMC7072059; doi:10.1007/s00405-020-05818-x)
Supplement: Supplementary file 1 — Supplementary file1 (DOCX 678 kb) [file 405_2020_5818_MOESM1_ESM.docx]

**Supplement Figure 1**. Positioning of the stimulation electrode at the three different sites: For stimulation of: A: Position 1 (P1) orbicularis oculi muscle; B: Position 2 (P2) zygomatic muscle; C: Position 3 (P3) orbicularis oris muscle.
