# Supplementary material for: Tolerability of facial electrostimulation in healthy adults and patients with facial synkinesis
Source: Eur Arch Otorhinolaryngol. 2020 Jan 24;277(4):1247–53. doi: 10.1007/s00405-020-05818-x (PMC7072059; doi:10.1007/s00405-020-05818-x)

**Supplement Figure 2**. Relation of discomfort to the stimulation amplitude. The ratio is increasing with longer pulse duration. A: motor threshold in the first study; B: motor threshold in the second study; C: tolerability threshold in the first study; D: tolerability threshold in the second study.


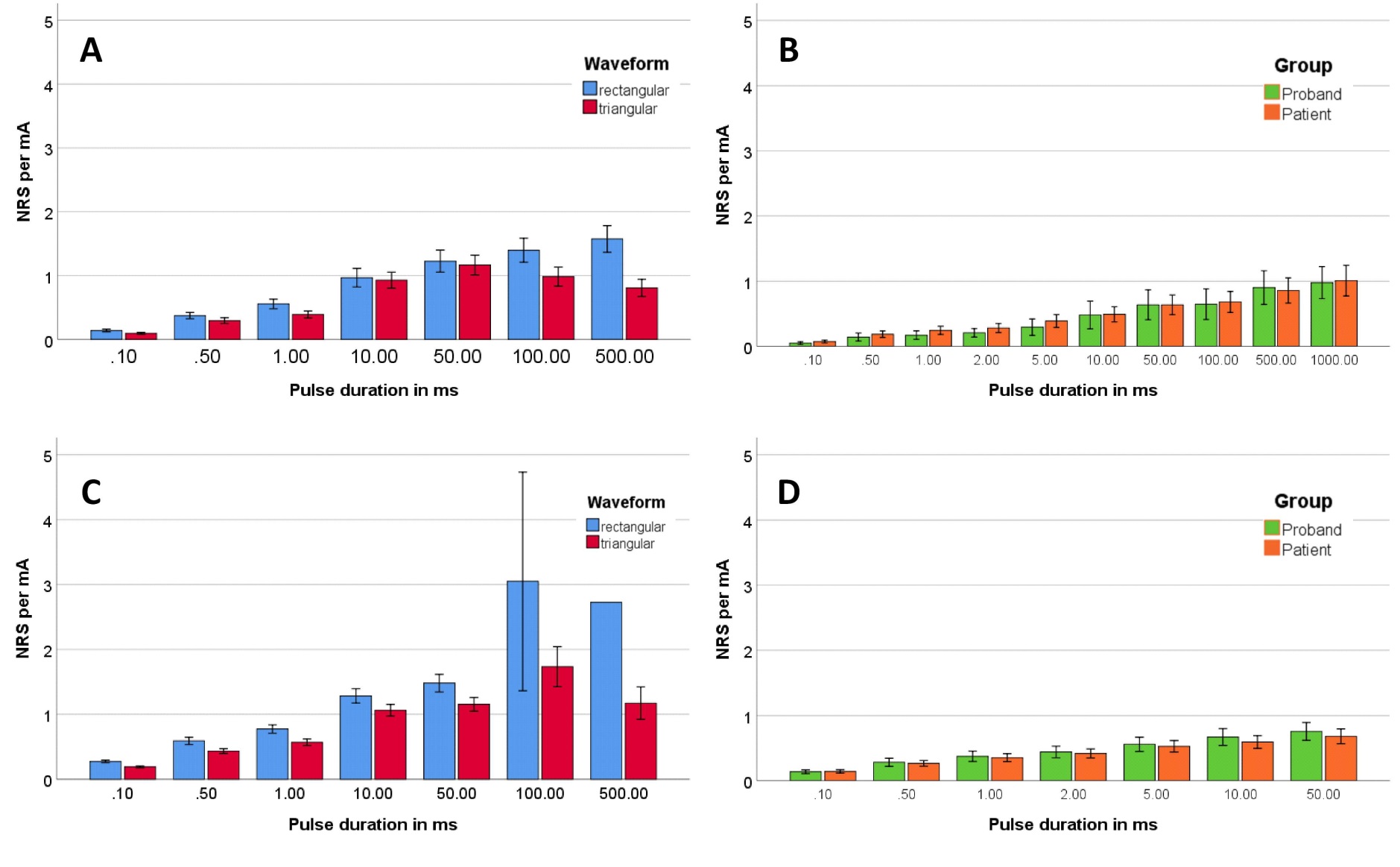

Supplement: Supplementary file 2 — Supplementary file2 (DOCX 238 kb) [file 405_2020_5818_MOESM2_ESM.docx]
