# Supplementary material for: Tolerability of facial electrostimulation in healthy adults and patients with facial synkinesis
Source: Eur Arch Otorhinolaryngol. 2020 Jan 24;277(4):1247–53. doi: 10.1007/s00405-020-05818-x (PMC7072059; doi:10.1007/s00405-020-05818-x)
Supplement: Supplementary file 3 — Supplementary file3 (DOCX 71 kb) [file 405_2020_5818_MOESM3_ESM.docx]

**Supplemental Tables**

**Supplemental Table 1**

| Supplemental Table 1.  First study: Comparison of the two pulse waveforms (rectangular and triangular) for facial electrostimulation in healthy probands (N=48). | | | | | | | | | | | | |  |
| --- | --- | --- | --- | --- | --- | --- | --- | --- | --- | --- | --- | --- | --- |
|  | Rectangular pulses | | | | | | Triangular pulses | | | | | |  |
| Puls duration | P1 | | P2 | | P3 | | P1 | | P2 | | P3 | |  |
| (ms) | Mean | SD | Mean | SD | Mean | SD | Mean | SD | Mean | SD | Mean | SD |  |
| Motor threshold (mA) | | | | | | | | | | | | | |
| 0.1 | 8.7 | 2.4 | 11.0 | 2.4 | 10.6 | 2.8 | 13.1 | 3.5 | 17.2 | 4.4 | 16.4 | 4.3 |  |
| 0.5 | 3.8 | 1.0 | 4.8 | 1.2 | 4.7 | 1.3 | 5.4 | 1.3 | 6.9 | 1.9 | 6.6 | 2.0 |  |
| 1 | 2.8 | 0.7 | 3.4 | 1.0 | 3.5 | 1.1 | 4.0 | 1.3 | 4.9 | 1.6 | 5.1 | 1.7 |  |
| 10 | 1.8 | 0.5 | 2.1 | 0.6 | 2.2 | 0.5 | 2.1 | 0.6 | 2.7 | 1.0 | 2.8 | 0.9 |  |
| 50 | 1.8 | 0.5 | 2.1 | 0.6 | 2.2 | 0.5 | 2.6 | 1.1 | 3.3 | 1.4 | 3.6 | 1.3 |  |
| 100 | 1.8 | 0.5 | 2.1 | 0.6 | 2.1 | 0.5 | 4.0 | 1.6 | 4.7 | 1.9 | 5.0 | 2.0 |  |
| 500 | 1.8 | 0.5 | 2.1 | 0.6 | 2.1 | 0.5 | 5.0 | 1.5 | 5.2 | 1.9 | 5.8 | 1.7 |  |
| Tolerability threshold (mA) | | | | | | | | | | | | | |
| 0.1 | 21.7 | 7.3 | 23.0 | 6.7 | 23.9 | 7.2 | 29.2 | 8.6 | 33.0 | 7.6 | 33.1 | 6.8 |  |
| 0.5 | 11.0 | 4.4 | 11.0 | 3.7 | 12.1 | 5.2 | 13.9 | 5.0 | 16.0 | 5.0 | 16.7 | 6.3 |  |
| 1 | 8.6 | 4.0 | 8.5 | 3.5 | 9.4 | 4.1 | 11.4 | 4.4 | 11.8 | 4.4 | 13.1 | 5.0 |  |
| 10 | 5.3 | 2.4 | 5.3 | 2.0 | 5.5 | 2.2 | 6.2 | 2.7 | 6.7 | 2.7 | 7.0 | 2.6 |  |
| 50 | 4.4 | 1.9 | 4.7 | 1.9 | 4.8 | 1.6 | 5.6 | 2.6 | 6.6 | 2.9 | 7.1 | 2.9 |  |
| 100 | NA | NA | 2.2 | NA | NA | NA | NA | 1.6 | 4.7 | 1.5 | 5.1 | 2.7 |  |
| 500 | NA | NA | 2.2 | NA | NA | NA | NA | 2.0 | 6.0 | 2.5 | 6.5 | 3.0 |  |
| Discomfort at motor threshold (NRS) | | | | | | | | | | | | | |
| 0.1 | 1.4 | 1.3 | 1.4 | 1.2 | 1.3 | 1.0 | 1.3 | 1.3 | 1.5 | 1.3 | 1.5 | 1.2 |  |
| 0.5 | 1.6 | 1.2 | 1.6 | 1.2 | 1.5 | 1.2 | 1.8 | 1.4 | 1.6 | 1.4 | 1.8 | 1.2 |  |
| 1 | 1.8 | 1.4 | 1.5 | 1.2 | 1.5 | 1.2 | 1.7 | 1.3 | 1.5 | 1.4 | 2.0 | 1.6 |  |
| 10 | 1.8 | 1.3 | 1.8 | 1.5 | 1.7 | 1.1 | 2.1 | 1.5 | 2.1 | 1.5 | 2.2 | 1.5 |  |
| 50 | 2.3 | 1.7 | 2.2 | 1.7 | 2.1 | 1.3 | 3.0 | 1.9 | 2.9 | 1.8 | 3.3 | 1.9 |  |
| 100 | 2.6 | 1.7 | 2.6 | 1.7 | 2.3 | 1.4 | 3.9 | 2.3 | 3.6 | 2.2 | 3.6 | 2.0 |  |
| 500 | 2.9 | 1.9 | 2.7 | 1.8 | 2.9 | 1.8 | 3.8 | 1.6 | 3.8 | 1.7 | 3.5 | 1.9 |  |
| Discomfort at tolerability threshold (NRS) | | | | | | | | | | | | | |
| 0.1 | 5.8 | 1.6 | 6.0 | 1.7 | 5.5 | 1.7 | 5.7 | 1.9 | 5.5 | 1.7 | 5.7 | 1.7 |  |
| 0.5 | 5.8 | 1.8 | 5.6 | 1.7 | 5.6 | 1.9 | 5.7 | 1.7 | 6.0 | 1.7 | 5.8 | 1.8 |  |
| 1 | 6.0 | 1.9 | 5.6 | 1.7 | 5.6 | 1.5 | 5.9 | 1.7 | 5.8 | 1.7 | 5.8 | 1.8 |  |
| 10 | 5.9 | 1.8 | 5.9 | 1.6 | 5.8 | 1.7 | 6.0 | 1.7 | 6.0 | 1.7 | 6.0 | 1.6 |  |
| 50 | 5.9 | 1.8 | 5.7 | 1.7 | 5.9 | 1.5 | 5.9 | 1.6 | 6.1 | 1.7 | 6.2 | 1.6 |  |
| 100 | NA | NA | 7.0 | NA | 7.0 | NA | 7.0 | 1.6 | 6.9 | 1.5 | 5.7 | 1.4 |  |
| 500 | NA | NA | 6.0 | NA | NA | NA | 5.9 | 2.4 | 5.5 | 2.1 | 6.4 | 2.8 |  |

NRS = numeric rating scale, NA = not applicable (maximal threshold not reached); P1/P2/P3 = three different electrostimulation sites in the face.

**Supplemental Table 2**

| Supplemental TABLE 2. First study: Felt side effects of electrostimulation. Comparison of the two waveforms (N=48). | | | | | | | | | | | | | | | | | |
| --- | --- | --- | --- | --- | --- | --- | --- | --- | --- | --- | --- | --- | --- | --- | --- | --- | --- |
|  |  | Rectangular pulses in ms | | | | | | | | Triangular pulses in ms | | | | | | | |
| Side effect | Position | Sum | 0.1 | 0.5 | 1 | 10 | 50 | 100 | 500 | Sum | 0.1 | 0.5 | 1 | 10 | 50 | 100 | 500 |
| light flash | P1 | 161 | 8 | 9 | 15 | 27 | 31 | 34 | 37 | 172 | 7 | 11 | 12 | 32 | 42 | 41 | 27 |
| light flash | P2 | 128 | 2 | 4 | 9 | 23 | 26 | 33 | 31 | 117 | 2 | 5 | 5 | 21 | 31 | 32 | 21 |
| light flash | P3 | 80 | 1 | 2 | 3 | 16 | 20 | 18 | 20 | 104 | 0 | 1 | 5 | 13 | 27 | 32 | 26 |
| light flash | Sum P1-P3 | 369 | 11 | 15 | 27 | 66 | 77 | 85 | 88 | 393 | 9 | 17 | 22 | 66 | 100 | 105 | 74 |
| masseter muscle contraction | P1 | 8 | 4 | 2 | 0 | 1 | 1 | 0 | 0 | 13 | 5 | 3 | 3 | 1 | 1 | 0 | 0 |
| masseter muscle contraction | P2 | 37 | 13 | 11 | 8 | 3 | 2 | 0 | 0 | 39 | 13 | 12 | 9 | 2 | 0 | 1 | 2 |
| masseter muscle contraction | P3 | 43 | 15 | 13 | 8 | 3 | 2 | 1 | 1 | 42 | 13 | 15 | 8 | 3 | 2 | 0 | 1 |
| masseter muscle contraction | Sum P1-P3 | 88 | 32 | 26 | 16 | 7 | 5 | 1 | 1 | 94 | 31 | 30 | 20 | 6 | 3 | 1 | 3 |
| toothaches | P1 | 11 | 3 | 3 | 3 | 0 | 0 | 1 | 1 | 14 | 3 | 5 | 3 | 0 | 0 | 1 | 2 |
| toothaches | P2 | 26 | 12 | 5 | 7 | 2 | 0 | 0 | 0 | 25 | 7 | 7 | 4 | 2 | 3 | 1 | 1 |
| toothaches | P3 | 16 | 5 | 4 | 2 | 0 | 2 | 2 | 1 | 22 | 7 | 6 | 3 | 3 | 1 | 0 | 2 |
| toothaches | Sum P1-P3 | 53 | 20 | 12 | 12 | 2 | 2 | 3 | 2 | 61 | 17 | 18 | 10 | 5 | 4 | 2 | 5 |
| metallic taste | P1 | 13 | 0 | 0 | 1 | 1 | 1 | 2 | 8 | 10 | 0 | 0 | 0 | 0 | 1 | 3 | 6 |
| metallic taste | P2 | 13 | 0 | 0 | 0 | 1 | 2 | 4 | 6 | 8 | 0 | 0 | 0 | 0 | 2 | 2 | 4 |
| metallic taste | P3 | 14 | 0 | 0 | 0 | 0 | 3 | 3 | 8 | 15 | 0 | 0 | 1 | 1 | 4 | 2 | 7 |
| metallic taste | Sum P1-P3 | 40 | 0 | 0 | 1 | 2 | 6 | 9 | 22 | 33 | 0 | 0 | 1 | 1 | 7 | 7 | 17 |
| shoulder muscle contraction | P1 | 10 | 4 | 3 | 2 | 1 | 0 | 0 | 0 | 5 | 2 | 1 | 1 | 0 | 0 | 0 | 1 |
| shoulder muscle contraction | P2 | 9 | 2 | 2 | 1 | 2 | 2 | 0 | 0 | 11 | 3 | 5 | 3 | 0 | 0 | 0 | 0 |
| shoulder muscle contraction | P3 | 20 | 5 | 4 | 5 | 2 | 2 | 0 | 2 | 15 | 4 | 4 | 4 | 2 | 1 | 0 | 0 |
| shoulder muscle contraction | Sum P1-P3 | 39 | 11 | 9 | 8 | 5 | 4 | 0 | 2 | 31 | 9 | 10 | 8 | 2 | 1 | 0 | 1 |

**Supplemental Table 3**

| Supplemental Table 3.  Second study: Comparison of facial electrostimulation using rectangular pulses in healthy probands and patients with postparetic facial palsy. | | | | | | | | | | | | |  |
| --- | --- | --- | --- | --- | --- | --- | --- | --- | --- | --- | --- | --- | --- |
|  | Healthy probands (N=30) | | | | | | Patients with postparetic facial palsy (N=30) | | | | | |  |
| Puls duration | P1 | | P2 | | P3 | | P1 | | P2 | | P3 | |  |
| (ms) | Mean | SD | Mean | SD | Mean | SD | Mean | SD | Mean | SD | Mean | SD |  |
| Motor threshold (mA) | | | | | | | | | | | | | |
| 0.1 | 9.2 | 2.7 | 11.2 | 3.6 | 11.1 | 4.1 | 14.8 | 7.2 | 15.9 | 6.5 | 15.4 | 6.7 |  |
| 0.5 | 3.8 | 1.2 | 5.0 | 1.8 | 4.9 | 2.0 | 7.1 | 4.2 | 7.3 | 3.4 | 7.6 | 3.9 |  |
| 1 | 3.0 | 0.9 | 3.7 | 1.5 | 3.7 | 1.6 | 5.2 | 3.1 | 5.4 | 2.6 | 6.3 | 3.8 |  |
| 2 | 2.4 | 0.8 | 3.0 | 1.2 | 3.1 | 1.2 | 4.4 | 3.0 | 4.5 | 2.4 | 5.0 | 2.8 |  |
| 5 | 2.0 | 0.7 | 2.3 | 1.1 | 2.6 | 1.1 | 3.5 | 2.2 | 3.6 | 2.3 | 4.1 | 2.3 |  |
| 10 | 1.7 | 0.8 | 2.1 | 1.0 | 2.4 | 1.0 | 3.0 | 1.6 | 3.2 | 2.3 | 3.7 | 2.0 |  |
| 50 | 1.7 | 0.8 | 2.1 | 1.0 | 2.2 | 0.9 | 2.7 | 1.4 | 3.0 | 2.0 | 3.4 | 1.7 |  |
| 100 | 1.7 | 0.8 | 2.0 | 1.0 | 2.2 | 0.8 | 2.6 | 1.2 | 2.8 | 1.9 | 3.2 | 1.6 |  |
| 500 | 1.7 | 0.8 | 1.9 | 1.0 | 2.2 | 0.9 | 2.6 | 1.2 | 2.8 | 1.8 | 3.3 | 1.6 |  |
| 1000 | 1.7 | 0.8 | 2.0 | 0.9 | 2.3 | 0.9 | 2.6 | 1.2 | 2.8 | 1.8 | 3.4 | 1.6 |  |
| Tolerability threshold (mA) | | | | | | | | | | | | | |
| 0.1 | 24.5 | 8.4 | 26.6 | 8.8 | 28.4 | 8.8 | 28.4 | 9.0 | 29.6 | 8.4 | 30.4 | 8.1 |  |
| 0.5 | 12.3 | 5.3 | 13.8 | 5.3 | 15.8 | 6.8 | 16.2 | 6.8 | 17.6 | 7.6 | 19.1 | 9.4 |  |
| 1 | 9.8 | 4.3 | 10.8 | 4.3 | 12.8 | 5.9 | 12.6 | 5.5 | 13.9 | 5.8 | 14.9 | 7.0 |  |
| 2 | 8.1 | 3.6 | 8.9 | 3.6 | 11.1 | 5.0 | 10.5 | 4.9 | 11.5 | 5.5 | 12.1 | 6.1 |  |
| 5 | 6.4 | 2.4 | 6.9 | 2.8 | 7.8 | 3.6 | 8.7 | 3.8 | 9.2 | 4.4 | 9.9 | 5.2 |  |
| 10 | 5.4 | 2.3 | 5.8 | 2.2 | 6.5 | 2.7 | 7.3 | 3.1 | 8.0 | 3.8 | 9.1 | 4.6 |  |
| 50 | 5.0 | 2.2 | 5.1 | 2.2 | 5.7 | 2.2 | 6.5 | 2.8 | 7.0 | 3.5 | 7.9 | 4.0 |  |
| 100 | NA | NA | NA | NA | NA | NA | NA | NA | NA | NA | NA | NA |  |
| 500 | NA | NA | NA | NA | NA | NA | NA | NA | NA | NA | NA | NA |  |
| 1000 | NA | NA | NA | NA | NA | NA | NA | NA | NA | NA | NA | NA |  |
| Discomfort at motor threshold (NRS) | | | | | | | | | | | | | |
| 0.1 | 0.4 | 0.8 | 0.4 | 0.9 | 0.5 | 0.9 | 1.2 | 1.8 | 1.2 | 1.5 | 0.8 | 1.2 |  |
| 0.5 | 0.4 | 0.7 | 0.6 | 0.9 | 0.7 | 1.3 | 1.1 | 1.6 | 1.5 | 1.8 | 1.2 | 1.7 |  |
| 1 | 0.4 | 0.6 | 0.5 | 0.7 | 0.6 | 0.9 | 1.3 | 1.7 | 1.4 | 1.5 | 1.2 | 1.7 |  |
| 2 | 0.4 | 0.7 | 0.6 | 0.8 | 0.6 | 0.8 | 1.2 | 1.6 | 1.4 | 1.6 | 1.0 | 1.4 |  |
| 5 | 0.4 | 0.7 | 0.6 | 0.7 | 0.6 | 0.7 | 1.4 | 1.8 | 1.4 | 1.8 | 1.2 | 1.4 |  |
| 10 | 0.5 | 0.8 | 0.8 | 1.0 | 0.7 | 0.9 | 1.5 | 1.9 | 1.6 | 1.6 | 1.5 | 1.9 |  |
| 50 | 0.7 | 0.9 | 1.0 | 1.3 | 1.2 | 1.6 | 1.7 | 1.9 | 2.0 | 2.0 | 1.5 | 1.7 |  |
| 100 | 0.6 | 0.9 | 1.1 | 1.5 | 1.2 | 1.6 | 1.7 | 1.9 | 1.9 | 1.9 | 1.7 | 1.9 |  |
| 500 | 1.2 | 1.2 | 1.3 | 1.7 | 1.4 | 1.5 | 2.5 | 2.4 | 2.2 | 2.5 | 2.1 | 2.2 |  |
| 1000 | 1.6 | 1.7 | 1.5 | 1.8 | 1.7 | 1.9 | 2.7 | 2.6 | 2.9 | 3.1 | 2.3 | 2.6 |  |
| Discomfort at tolerability threshold (NRS) | | | | | | | | | | | | | |
| 0.1 | 2.9 | 2.4 | 3.0 | 2.6 | 3.1 | 2.6 | 3.8 | 2.8 | 4.2 | 2.8 | 3.5 | 2.6 |  |
| 0.5 | 3.0 | 2.5 | 2.9 | 2.6 | 3.1 | 2.7 | 4.1 | 2.6 | 4.1 | 2.7 | 3.9 | 2.4 |  |
| 1 | 3.2 | 2.4 | 3.0 | 2.5 | 3.2 | 2.7 | 4.0 | 2.7 | 4.4 | 2.7 | 4.0 | 2.5 |  |
| 2 | 3.2 | 2.3 | 3.1 | 2.5 | 3.2 | 2.6 | 3.9 | 2.5 | 4.3 | 2.7 | 3.9 | 2.4 |  |
| 5 | 3.3 | 2.4 | 3.0 | 2.5 | 3.3 | 2.5 | 4.0 | 2.7 | 4.2 | 2.7 | 4.2 | 2.6 |  |
| 10 | 3.1 | 2.3 | 3.2 | 2.5 | 3.3 | 2.6 | 3.9 | 2.6 | 4.3 | 2.8 | 4.1 | 2.5 |  |
| 50 | 3.1 | 2.2 | 3.2 | 2.5 | 3.3 | 2.6 | 3.9 | 2.7 | 4.4 | 2.7 | 4.1 | 2.4 |  |
| 100 | NA | NA | NA | NA | NA | NA | NA | NA | NA | NA | NA | NA |  |
| 500 | NA | NA | NA | NA | NA | NA | NA | NA | NA | NA | NA | NA |  |
| 1000 | NA | NA | NA | NA | NA | NA | NA | NA | NA | NA | NA | NA |  |

NRS = numeric rating scale, NA = not applicable (maximal threshold not reached); P1/P2/P3 = three different electrostimulation sites in the face.

**Supplemental table 4**

| Supplemental TABLE 4 Second study: Felt side effects of electrostimulation. Comparison of healthy probands and patients with postparetic facial palsy. | | | | | | | | | | | | | | | | | | | | | | | |
| --- | --- | --- | --- | --- | --- | --- | --- | --- | --- | --- | --- | --- | --- | --- | --- | --- | --- | --- | --- | --- | --- | --- | --- |
|  |  | Healthy probands (N=30) | | | | | | | | | | | Patients with postparetic facial palsy. (N=30) | | | | | | | | | | |
|  |  | Rectangular pulses in ms | | | | | | | | | | | Rectangular pulses in ms | | | | | | | | | | |
| Side effect | Position | Sum | 0.1 | 0.5 | 1 | 2 | 5 | 10 | 50 | 100 | 500 | 1000 | Sum | 0.1 | 0.5 | 1 | 2 | 5 | 10 | 50 | 100 | 500 | 1000 |
| light flash | P1 | 140 | 5 | 5 | 5 | 9 | 10 | 13 | 16 | 26 | 25 | 26 | 142 | 5 | 4 | 8 | 12 | 11 | 18 | 19 | 20 | 23 | 22 |
| light flash | P2 | 98 | 2 | 2 | 3 | 6 | 8 | 9 | 13 | 17 | 19 | 19 | 87 | 4 | 3 | 5 | 6 | 7 | 11 | 13 | 12 | 14 | 12 |
| light flash | P3 | 39 | 1 | 1 | 1 | 1 | 3 | 4 | 6 | 5 | 9 | 8 | 48 | 1 | 1 | 3 | 4 | 4 | 5 | 6 | 9 | 8 | 7 |
| light flash | Sum P1-P3 | 277 | 8 | 8 | 9 | 16 | 21 | 26 | 35 | 48 | 53 | 53 | 277 | 10 | 8 | 16 | 22 | 22 | 34 | 38 | 41 | 45 | 41 |
| masseter muscle contraction | P1 | 5 | 0 | 0 | 0 | 0 | 0 | 0 | 0 | 0 | 2 | 3 | 9 | 0 | 0 | 0 | 0 | 0 | 0 | 0 | 1 | 5 | 3 |
| masseter muscle contraction | P2 | 13 | 0 | 0 | 0 | 0 | 0 | 0 | 0 | 2 | 5 | 6 | 11 | 0 | 0 | 0 | 0 | 0 | 0 | 0 | 1 | 9 | 1 |
| masseter muscle contraction | P3 | 10 | 0 | 0 | 0 | 0 | 0 | 0 | 0 | 2 | 4 | 4 | 10 | 0 | 0 | 0 | 1 | 0 | 0 | 0 | 1 | 3 | 5 |
| masseter muscle contraction | Sum P1-P3 | 28 | 0 | 0 | 0 | 0 | 0 | 0 | 0 | 4 | 11 | 13 | 30 | 0 | 0 | 0 | 1 | 0 | 0 | 0 | 3 | 17 | 9 |
| toothaches | P1 | 5 | 0 | 1 | 1 | 0 | 0 | 0 | 0 | 0 | 0 | 3 | 1 | 1 | 0 | 0 | 0 | 0 | 0 | 0 | 0 | 0 | 0 |
| toothaches | P2 | 7 | 2 | 1 | 1 | 1 | 0 | 0 | 0 | 0 | 1 | 1 | 23 | 8 | 3 | 2 | 1 | 2 | 0 | 2 | 1 | 2 | 2 |
| toothaches | P3 | 5 | 1 | 1 | 0 | 1 | 0 | 0 | 1 | 0 | 0 | 1 | 20 | 7 | 4 | 4 | 3 | 1 | 0 | 1 | 0 | 0 | 0 |
| toothaches | Sum P1-P3 | 17 | 3 | 3 | 2 | 2 | 0 | 0 | 1 | 0 | 1 | 5 | 44 | 16 | 7 | 6 | 4 | 3 | 0 | 3 | 1 | 2 | 2 |
| metallic taste | P1 | 14 | 0 | 0 | 0 | 1 | 1 | 0 | 2 | 1 | 5 | 4 | 26 | 0 | 0 | 1 | 1 | 1 | 0 | 2 | 2 | 9 | 10 |
| metallic taste | P2 | 15 | 0 | 0 | 0 | 0 | 1 | 1 | 1 | 2 | 5 | 5 | 28 | 0 | 0 | 0 | 0 | 1 | 1 | 2 | 2 | 11 | 11 |
| metallic taste | P3 | 19 | 1 | 1 | 0 | 0 | 0 | 0 | 1 | 1 | 8 | 7 | 28 | 0 | 1 | 0 | 0 | 0 | 0 | 2 | 3 | 10 | 12 |
| metallic taste | Sum P1-P3 | 48 | 1 | 1 | 0 | 1 | 2 | 1 | 4 | 4 | 18 | 16 | 82 | 0 | 1 | 1 | 1 | 2 | 1 | 6 | 7 | 30 | 33 |
| shoulder muscle contraction | P1 | 0 | 0 | 0 | 0 | 0 | 0 | 0 | 0 | 0 | 1 | 1 | 4 | 0 | 2 | 2 | 0 | 0 | 0 | 0 | 0 | 0 | 0 |
| shoulder muscle contraction | P2 | 0 | 0 | 0 | 0 | 0 | 0 | 0 | 0 | 0 | 0 | 1 | 4 | 1 | 1 | 1 | 1 | 0 | 0 | 0 | 0 | 0 | 0 |
| shoulder muscle contraction | P3 | 0 | 0 | 0 | 0 | 0 | 0 | 0 | 0 | 0 | 1 | 1 | 2 | 0 | 0 | 0 | 2 | 0 | 0 | 0 | 0 | 0 | 0 |
| shoulder muscle contraction | Sum P1-P3 | 5 | 0 | 0 | 0 | 0 | 0 | 0 | 0 | 0 | 2 | 3 | 10 | 1 | 3 | 3 | 3 | 0 | 0 | 0 | 0 | 0 | 0 |
